# Tuning the Performance of Ge-Doped CZTSSe Solar Cells via Selenization

**DOI:** 10.3390/ma19071337

**Published:** 2026-03-27

**Authors:** Xiaogong Lv, Shumin Zhang, Yanchun Yang, Guonan Cui, Wenliang Fan, Xing Yue

**Affiliations:** 1 College of Information Engineering, Ordos Institute of Technology, Ordos 017000, China; nmfwl1982@126.com (W.F.); xingyue@oit.edu.cn (X.Y.); 2 Inner Mongolia Key Laboratory for Physics and Chemistry of Functional Materials, School of Physics and Electronic Information, Inner Mongolia Normal University, 81 Zhaowuda Road, Huhhot 010022, China; ordoslxg@126.com (S.Z.); 20236013006@mails.imnu.edu.cn (G.C.)

**Keywords:** kesterite solar cells, low-concentration Ge doping, CZTGSSe thin film, selenization temperature, process optimization, optoelectronic properties

## Abstract

Cu_2_ZnSn(S,Se)_4_ (CZTSSe) is a candidate thin-film photovoltaic material; however, its performance is restricted by innate defect-induced nonradiative recombination. Low-concentration Ge doping has been identified as an efficient way to mitigate these defects, but the selenization temperature remains an important process parameter that governs the structure and optoelectronic characteristics of CZTSSe absorbers. In the present work, low-concentration Ge-doped Cu_2_ZnSn_0.95_Ge_0.05_S_4_ (CZTGS) precursor films were synthesized through a green, n-butylammonium butyrate-based solution approach. The effects of the selenization temperature (530–570 °C) on the microstructure, composition, and photovoltaic performance of Cu_2_ZnSn_0.95_Ge_0.05_(S,Se)_4_ (CZTGSSe) films and devices were comprehensively investigated. X-ray diffraction (XRD), scanning electron microscopy (SEM), X-ray photoelectron spectroscopy (XPS), energy-dispersive X-ray spectrometer (EDS), atomic force microscopy (AFM) were performed to comprehensively characterize the synthesized samples, and the results suggested that the selenization temperature dramatically altered the film grain growth, crystallinity, elemental retention and surface roughness. Specifically, the film that underwent selenization at 550 °C presented the best crystallinity, which was accompanied by large-scale even grains, efficient Ge^4+^ addition to the kesterite lattice and the lowest surface roughness. These better properties in terms of structure and composition resulted in the lowest carrier transport resistance (*R*_s_ = 8.6 Ω∙cm^2^), improved recombination resistance (*R*_j_ = 5.9 kΩ∙cm^2^), inhibited nonradiative recombination, and prolonged carrier lifetime (*τ*_EIS_ = 35.8 μs). Therefore, the resulting CZTGSSe thin-film solar cell had an 8.69% better power conversion efficiency (PCE), while its open-circuit voltage (*V*_OC_) was 0.42 V, the fill factor (FF) was 55.51%, and the short-circuit current density (*J*_SC_) was 37.71 mA·cm^−2^. Our results elucidate the mechanism by which the selenization temperature regulates low-concentration Ge-doped kesterite devices and provide more insights into the optimization of processes for cost-effective, high-performance, and green thin-film solar cells.

## 1. Introduction

Owing to their immense potential for low-cost, high-efficiency energy conversion, next-generation photovoltaic technologies have become a focal point of research on sustainable energy amid the global push for carbon neutrality [1]. Among the various competing technologies, CuIn_1−*x*_Ga*_x_*Se_2_ (CIGS) and CdTe thin-film solar cells have achieved high power conversion efficiencies [2]. However, they are constrained by the limited supply of rare elements such as In and Ga and the toxicity of Cd. III–V semiconductor nanowire cells exhibit excellent optoelectronic properties and strong light absorption capabilities, yet their adoption is hindered by complex fabrication processes and high costs [3,4]. Organic–inorganic hybrid perovskite cells, with the highest certified power conversion efficiency exceeding 33%, offer significant cost advantages but face challenges in terms of long-term operational stability [5]. In contrast, Cu_2_ZnSn(S,Se)_4_ (CZTSSe) has emerged as a novel chalcopyrite-type semiconductor material. Composed of earth-abundant elements, it features environmentally friendly and favorable optoelectronic characteristics. Directly inheriting the device architecture of CIGS has the potential to overcome the resource bottlenecks associated with CIGS, thereby attracting widespread attention in the photovoltaic community [1,6,7]. In recent years, significant progress has been made in CZTSSe thin-film solar cell research, with laboratory-scale small-area devices achieving power conversion efficiencies exceeding 15% [8]. However, this efficiency still lags behind that of commercially mature materials such as CIGS and CdTe and remains far below the theoretical Shockley–Queisser limit of about 32% for single-junction devices [9]. Research has indicated that non-radiative recombination, primarily induced by complex defect energy levels and band tail states within the CZTSSe material, constitutes the core bottleneck restricting further performance improvements [10,11].

Cation alloying, which involves the partial substitution of elements such as Cu, Zn, or Sn with other elements from the same group (e.g., Ag for Cu, Cd for Zn, and Ge for Sn), is an effective strategy for inhibiting antisite defects and reducing the open-circuit voltage deficit (*V*_OC,def_) of CZTSSe solar cells at the compositional level [12]. Among the numerous substitutional elements, the replacement of Sn by the Group IV element Ge results in outstanding performance, and low-proportion Ge doping has unique advantages: first, the introduction of Ge^4+^ can broaden the bandgap by increasing the conduction band minimum (CBM) and optimizing the absorber layer-buffer layer band alignment [13]; second, Ge only stably exists in the +4 valence state in compounds, which can effectively eliminate Sn^2+^-related deep-level defects [14]; third, the GeS(Se)_2_ phase formed during selenization can act as a flux to promote grain growth and improve film crystallinity [15]; and fourth, low-concentration doping can avoid the reduction in the light absorption range, deterioration of the band structure, and precipitation of secondary phases caused by high Ge content [16]. On this basis, our research group previously conducted low-concentration doping studies with Ge/(Ge + Sn) ratios ranging from 0 to 0.2, confirming that when the doping ratio is 0.05, the device performance is significantly improved, and this ratio is the optimal doping ratio for this system—which is consistent with recent reports on Ge-doped kesterite solar cells where moderate Ge incorporation (Ge/(Ge + Sn) = 0.03–0.07) yields the best balance between defect suppression and band structure optimization [17].

The selenization temperature is an important process parameter related to the fabrication of high-performance CZTSSe solar cells, and it directly affects the absorber layer in terms of its morphological features, crystal structure, defect state and elemental distribution [18,19]. A suitable selenization temperature contributes to promoting adequate grain growth and reducing the defect density, thus optimizing the recombination dynamics and carrier transport [20], but too high or too low of a temperature results in elemental volatilization, film decomposition, inadequate crystallization, and severely deteriorated device performance. Many studies have been conducted to explore low-proportion Ge doping [12,16], and they have focused mainly on optimizing the Ge-doping ratio; however, systematic and in-depth studies comprehensively investigating the synergistic regulatory effects of the selenization temperature on the microstructure, photovoltaic features, carrier dynamics and elemental retention of CZTGSSe absorber layers at the optimal Ge-doping ratio are still lacking and have emerged as key limiting factors for further improvement of device performance. Moreover, most of the existing preparation methods for Ge-doped CZTGSSe precursor films either use toxic organic solvents or involve complex process steps, greatly limiting their industrial application potential. The development of a green, low-cost and scalable solution preparation method combined with precise process parameter optimization is therefore highly practical for the commercialization of kesterite solar cells.

By utilizing a green and cost-effective n-butylammonium butyrate-based solution approach that might be adopted for industrial applications, the present work synthesized low-concentration Ge-doped (*x* = 0.05) CZTGS precursor films. Thereafter, we comprehensively explored how selenization temperatures within 530–570 °C affected the absorber layer morphology, microstructure, elemental composition, and associated photovoltaic device performance. According to our findings, the selenization temperature significantly regulated the grain size, crystallinity, elemental retention and surface roughness of the CZTGSSe films. Specifically, the CZTGSSe film synthesized at 550 °C had the best crystalline structure and morphology, decreased recombination loss and reduced carrier transport resistance. Finally, the maximum PCE of the obtained CZTGSSe thin-film solar cell was 8.69% at the best selenization temperature (550 °C). Our results not only elucidate the process optimization for low-concentration Ge-doped CZTSSe solar cells but also provide important experimental support and theoretical guidance for the development of green, cost-effective and high-performance kesterite thin-film photovoltaic devices.

## 2. Materials and Methods

### 2.1. Materials and Precursor Solution Preparation

Our chemical reagents, such as ZnO (Aladdin Biochemical Technology Co., Ltd., Shanghai, China, 99.99%), copper acetate monohydrate (ibid, 99.95%), GeO_2_ (ibid, 99.9%), SnCl_2_·2H_2_O (ibid, 99.99%), selenium powder (ibid, 99.9%), thiourea (Sinopharm Chemical Reagent Co., Ltd., Shanghai, China, 99%), and other solvents (n-butylamine, butyric acid, ethanolamine, thioglycolic acid, ethanol, 2-methoxyethanol, ibid), were utilized with no additional purification.

A 1 mmol/mL GeO_2_ precursor solution comprising thioglycolic acid (1 mL), 2-methoxyethanol (2 mL), and ethanolamine (1 mL) was synthesized by adding 4 mmol of GeO_2_ to the sample prior to 24 h of stirring at 60 °C. Later, 1.7 mmol of copper acetate monohydrate alongside 1.2 mmol of ZnO was added to the mixture containing n-butylamine and butyric acid (2 mL each) at 120 °C. After SnCl_2_·2H_2_O (0.95 mmol) was added with brief stirring, the solution was cooled. Thiourea (5 mmol) and ethanol (6 mL) were then introduced, yielding a clear CZTS solution after centrifugation (12,000 rpm, 5 min). Finally, 0.05 mL of the GeO_2_ precursor was incorporated to obtain the CZTGS precursor solution.

### 2.2. Fabrication of CZTGSSe Solar Cells

The CZTGS precursor film was deposited onto Mo-coated soda–lime glass substrates (GULUO Glass Co., Ltd., Luoyang, China) by spin-coating, followed by thermal curing of each layer at 320 °C for 2 min, all of which were conducted under the same high-purity nitrogen atmosphere. This coating-curing cycle was repeated nine times under ambient conditions to construct a precursor film with a thickness of ~1.4 μm, which was measured using a stylus profilometer (Bruker Dektak XT, Bruker Corporation, Billerica, MA, USA). The films were subsequently selenized in a high-purity nitrogen atmosphere via a two-step annealing process: initially at 400 °C for 10 min, followed by a 10-min high-temperature treatment at 530–570 °C, yielding the final CZTGSSe absorbers.

**Figure 1:**
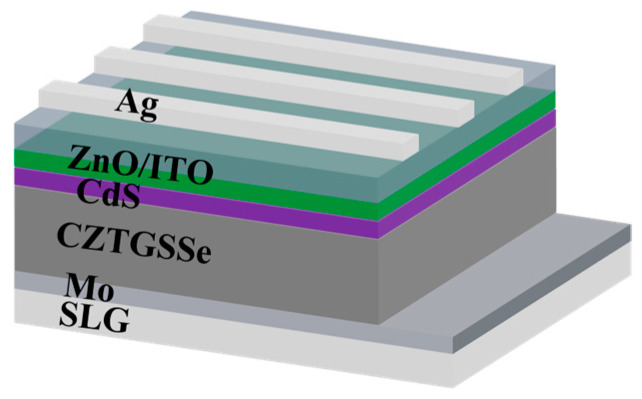
Schematic of the CZTGSSe solar cell.

The completed solar cells had a standard SLG/Mo/CZTGSSe/CdS/ZnO/ITO/Ag structure, as shown in Figure 1. Through chemical bath deposition, we deposited an ~40 nm thick CdS buffer layer and dried it at 120 °C. A bilayer transparent conducting oxide (50 nm i-ZnO alongside 170 nm ITO) was subsequently sputtered, followed by the thermal evaporation of Ag grid electrodes (~200 nm). Individual devices whose active area was ~0.19 cm^2^ were then defined by mechanical scribing.

### 2.3. Device and Film Characterization

The film crystalline phase was characterized by X-ray diffraction (XRD, Bruker D8 Focus, Bruker Corporation, Billerica, MA, USA) with Cu Kα radiation (λ = 1.5406 Å), a scanning range of 10–80°, a step size of 0.02°, and a scanning speed of 5°·min^−1^. The surface and cross-sectional morphologies were observed by scanning electron microscopy (SEM, Hitachi S-4800, Hitachi High-Technologies Corporation, Tokyo, Japan) with an accelerating voltage of 5–10 kV, a working distance of 8–10 mm, and secondary electron (SE) detection mode; the elemental composition was analyzed via an attached energy-dispersive X-ray spectrometer (EDS, Bruker AXS XFlash 4010, Bruker Corporation, Billerica, MA, USA) with an acquisition time of 60 s and a probe current of 1 nA. X-ray photoelectron spectroscopy (XPS, ESCALAB-MKII 250, Thermo Fisher Scientific Inc., Waltham, MA, USA) was carried out to analyze the chemical states with an Al Kα source (hν = 1486.6 eV) under a vacuum pressure < 1 × 10^−9^ mbar; atomic force microscopy (AFM, Bruker Dimension Icon, Bruker Corporation, Billerica, MA, USA) was used to evaluate surface roughness and topography in tapping mode with a scan rate of 1 Hz and a scan area of 5 μm × 5 μm; the photovoltaic performance was assessed by measuring current density–voltage (*J–V*) curves upon illumination at AM 1.5G (100 mW·cm^−2^) with a T-SCSpec IV station (Newport Corporation, Irvine, CA, USA, data acquisition software version: v3.2.1), using a scan voltage range of 0–1.0 V and a scan rate of 0.01 V·s^−1^; and electrical impedance spectroscopy (EIS) measurements were carried out away from light at zero bias with a Chi660e electrochemical workstation (Shanghai Chenhua Instrument Co., Ltd., Shanghai, China, test software version: Chi660e v4.11) over a frequency range of 0.1 Hz–10^5^ Hz and an AC perturbation amplitude of 10 mV. A Newport QuantX-300 system (Newport Corporation, Irvine, CA, USA, software version: QuantX v2.5.0) was used to obtain external quantum efficiency (EQE) spectra with a wavelength range of 300–1100 nm, a step size of 10 nm, and a bias voltage of 0 V. Modulated transient photovoltage (M-TPV) measurements were carried out using a 532-nm pulsed laser (Brio, Laser Quantum Ltd., Stockport, UK, 10 Hz, 4 ns) for excitation and a Tektronix DPO 7104 digital oscilloscope (Tektronix, Inc., Beaverton, OR, USA, input impedance: 1 MΩ; software version: TekScope v5.1.0) to record the photovoltage decay process.

## 3. Results

**Figure 2:**
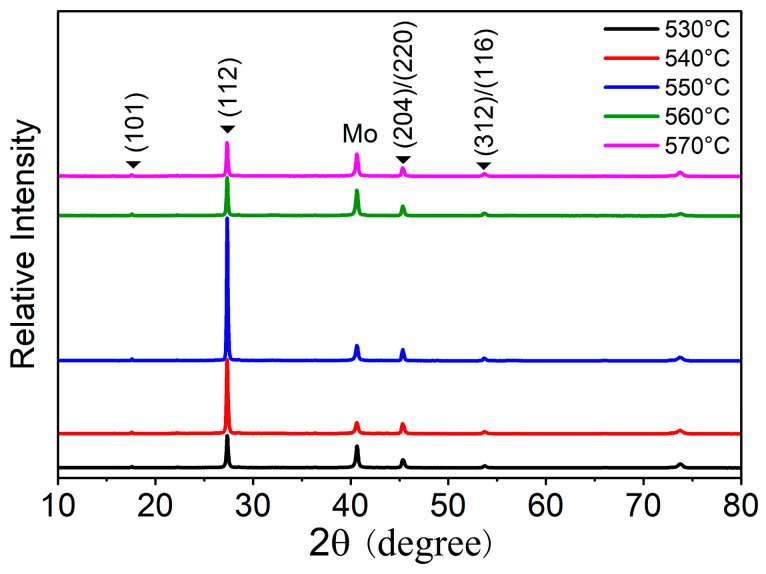
XRD patterns of CZTGSSe films selenized at different selenization temperatures.

The XRD patterns of the CZTGSSe films selenized at different temperatures are shown in Figure 2. Every diffraction maximum is associated with the kesterite structure (space group I4^−^), with the major reflections corresponding to the (112), (204)/(220), and (312)/(116) planes, confirming the formation of a pure phase without detectable secondary compounds [21]. By fitting the dominant diffraction peak of the (112) crystal plane in the XRD curve, the full width at half maximum (FWHM) values of the CZTGSSe thin films selenized at 530–570 °C were calculated to be 0.217, 0.197, 0.157, 0.170, and 0.177. Before 550 °C, the continuous decrease in the FWHM indicates that the crystallinity of the thin films is improved and that the grain size is increased. Among them, the FWHM of the (112) crystal plane is the smallest at 550 °C, confirming that the thin film has the best crystalline quality at this temperature. This finding also verifies the importance of optimizing the selenization process to improve the efficiency of kesterite solar cells [22]. The texture coefficient *T_c_*(*hkl*) is also used to evaluate the degree of preferred crystal growth orientation of the (112) crystal plane in CZTGSSe thin films, and its value can be calculated from XRD data using the following formula [23]:

(1) $$T_{c}(hkl)=\frac{I(hkl)/I_{0}(hkl)}{N^{-1}\sum I(hkl)/I_{0}(hkl)}$$

where *T_c_*(*hkl*), *I*(*hkl*), *N*, and (*hkl*) represent the texture coefficient of the crystal plane, measured intensity, number of diffraction peaks, and Miller indices, respectively, and *I*_0_(*hkl*) is taken from the measured XRD intensity of the CZTGSSe powder. The *T_c_*(112) values of the thin films selenized at 530–570 °C are 2.289, 3.296, 3.891, 2.806, and 2.314, respectively. The texture coefficient first tends to increase but then decreases, reaching a maximum at 550 °C. This finding indicates that the selenization temperature regulates the preferential growth of CZTGSSe thin films along the (112) orientation—an energetically favorable direction for charge migration in kesterite materials. Specifically, an increase in *T_c_*(112) enhances the uniformity of grain orientation and reduces grain boundary misalignment and defect accumulation at grain boundaries, thereby minimizing carrier scattering and trapping during transport to improve carrier transmission efficiency [24]. Moreover, the enhanced preferred orientation contributes to the improved crystallinity of the CZTGSSe thin film, which is consistent with the above FWHM results. From the above observations, the selenization temperature accounts for an essential parameter that governs the grain growth and crystallinity of CZTGSSe films, and 550 °C was the best condition for obtaining a well-organized crystal structure.

**Figure 3:**
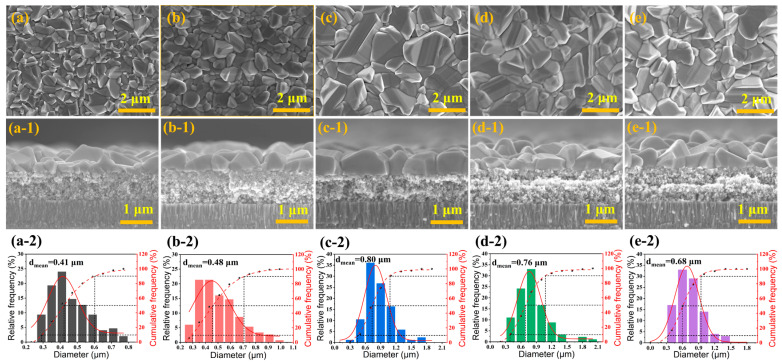
Planar-view (**a**–**e**) and cross-sectional (**a-1**–**e-1**) SEM micrographs and particle size distribution histograms (**a-2**–**e-2**) of CZTGSSe films selenized at 530 °C, 540 °C, 550 °C, 560 °C, and 570 °C.

Planar-view (a–e) and cross-sectional (a-1–e-1) SEM images and particle size distribution histograms (a-2–e-2) of CZTGSSe films selenized at 530–570 °C are shown in Figure 3. These films exhibit a representative bilayer structure, comprising a large-grained, dense upper layer and a fine-grained lower layer—a feature most likely attributed to restricted selenium vapor diffusion during the selenization process [15]. SEM observations reveal that the grain size of the upper layer gradually increases and that the surface morphology becomes more uniform and compact as the selenization temperature increases from 530 °C to 550 °C, a trend verified by statistical analysis of grain size distributions. The average grain sizes are 0.41, 0.48, and 0.80 μm at 530 °C, 540 °C, and 550 °C, respectively, and the narrowest grain size distribution occurs at 550 °C, thus indicating that the most uniform grain morphology occurs at this temperature. These results confirm that increasing the selenization temperature facilitates enhanced grain growth while reducing the grain boundary density. Simultaneously, the thickness of the large-grained layer increases significantly, whereas that of the fine-grained layer decreases—a structural evolution that effectively improves charge transport and suppresses grain boundary recombination. Notably, when the temperature exceeds 550 °C, the grain size of the upper layer begins to decrease gradually, and the surface uniformity deteriorates slightly, as further validated by the statistical analysis of grain size distributions, with the average grain sizes being 0.76 and 0.68 μm at 560 °C and 570 °C, respectively. This phenomenon is likely attributed to the partial decomposition of the films and the enhanced volatilization of Ge-containing species at elevated temperatures. According to the above findings, the best selenization temperature of 550 °C results in CZTGSSe films with a good microstructure, featuring a thickened compact layer, large-scale grains, and few morphological defects.

**Figure 4:**
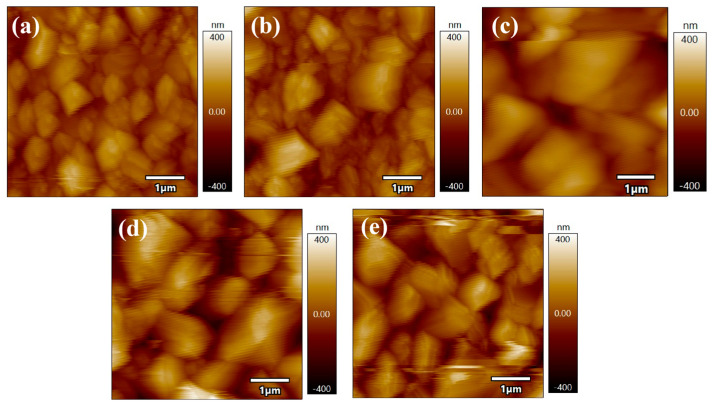
AFM surface images (5 μm × 5 μm area) of CZTGSSe films selenized at (**a**) 530 °C, (**b**) 540 °C, (**c**) 550 °C, (**d**) 560 °C, and (**e**) 570 °C.

**Figure 5:**
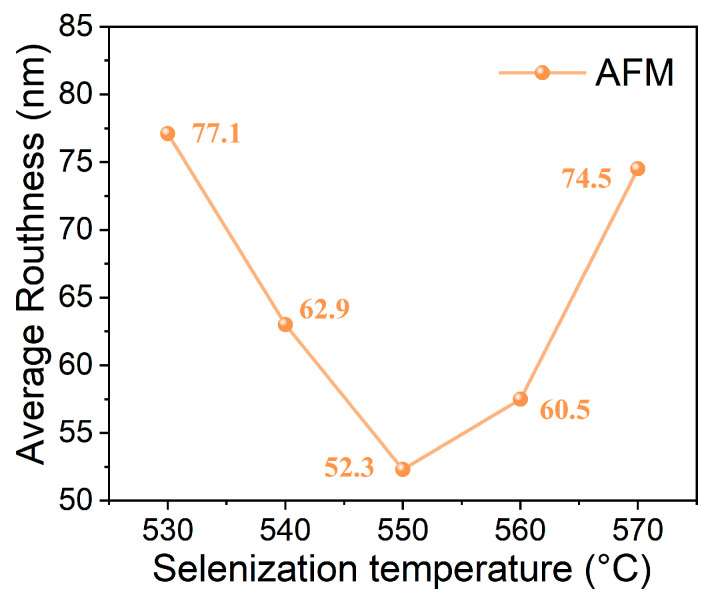
Average roughness (nm) of CZTGSSe thin films as a function of selenization temperature (530–570 °C).

The AFM images (5 μm × 5 μm) of the CZTGSSe thin films selenized at various temperatures are shown in Figure 4, and the surface roughness as a function of the selenization temperature is plotted in Figure 5. The average surface roughness of the CZTGSSe thin films first decreases but then increases with increasing selenization temperature. Specifically, it decreases from 77.1 nm at 530 °C to a minimum of 52.3 nm at 550 °C—a trend ascribed to grain coalescence and growth, which renders the film surface smoother and denser. This improved morphology enhances the buffer layer coverage, reduces interfacial defects, and ultimately increases the device performance, as previously reported for kesterite solar cells [20]. Subsequently, the average roughness increases again to 60.5 nm at 560 °C and 74.5 nm at 570 °C, which is likely due to surface degradation at elevated temperatures. Furthermore, the AFM images reveal that the grain size of the CZTGSSe thin films also tends to first increase but then decrease with increasing selenization temperature, with the largest grain size occurring at 550 °C. These findings are in good agreement with the SEM observations.

**Figure 6:**
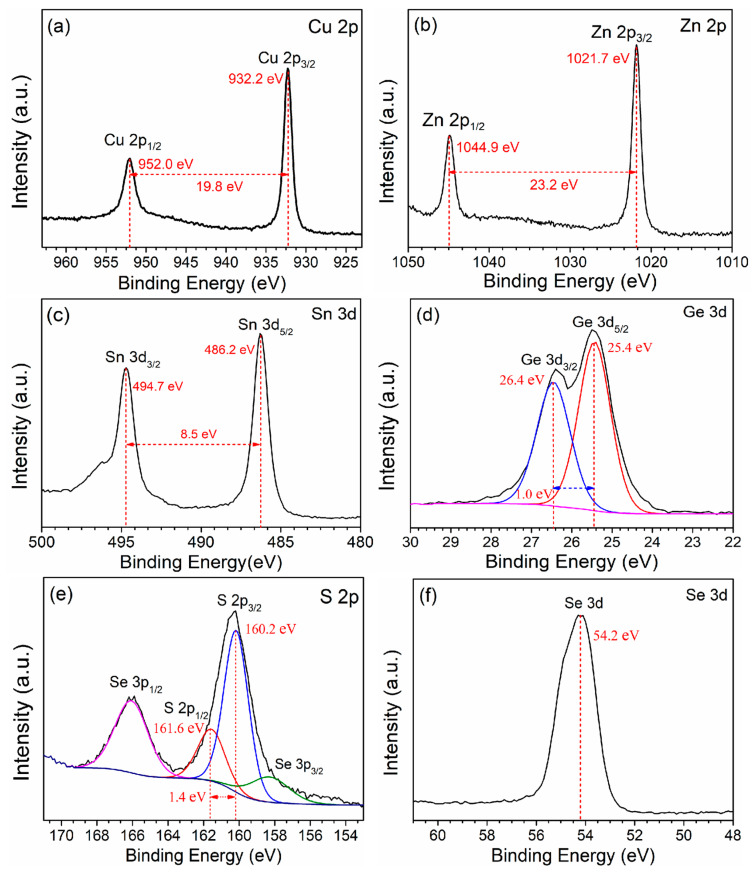
XPS characterization of the CZTGSSe film selenized at 550 °C showing the core-level spectra for (**a**) Cu 2p, (**b**) Zn 2p, (**c**) Sn 3d, (**d**) Ge 3d, (**e**) S 2p, and (**f**) Se 3d.

XPS was employed to analyze the chemical states and valence configurations of the elements in the CZTGSSe film selenized at 550 °C (Figure 6). For the Cu 2p core-level spectrum (Figure 6a), two distinct spin–orbit splitting peaks are observed at 932.2 and 952.0 eV (Cu 2p_3/2_ and Cu 2p_1/2_, respectively), and the energy separation is 19.8 eV, corresponding to the Cu^+^ valence state. The Zn 2p spectrum (Figure 6b) exhibits a doublet at 1021.7 and 1044.9 eV (Zn 2p_3/2_ and Zn 2p_1/2,_ respectively), and the splitting energy is 23.2 eV, confirming the presence of Zn^2+^. In the Sn 3d region (Figure 6c), two prominent peaks were located at 486.2 and 494.7 eV (Sn 3d_5/2_ and Sn 3d_3/2_), and the splitting was 8.5 eV, which is characteristic of Sn^4+^. The Ge 3d core-level spectrum (Figure 6d) shows two well-resolved peaks at 25.4 and 26.4 eV (Ge 3d_5/2_ and Ge 3d_3/2_) isolated by 1.0 eV, verifying that Ge^4+^ is successfully incorporated into the kesterite lattice via substitution for Sn^4+^. In the S 2p spectrum (Figure 6e), the deconvoluted peaks at 160.2 and 161.6 eV (S 2p_3/2_ and S 2p_1/2,_ respectively) have a 1.4 eV energy difference and are assigned to S^2−^. In the Se 3d spectrum (Figure 6f), the peak centered at 54.2 eV corresponds to Se^2−^. All the experimental results are consistent with previously reported values [15,25]. Together, these XPS results confirm the formation of CZTGSSe.

**Figure 7:**
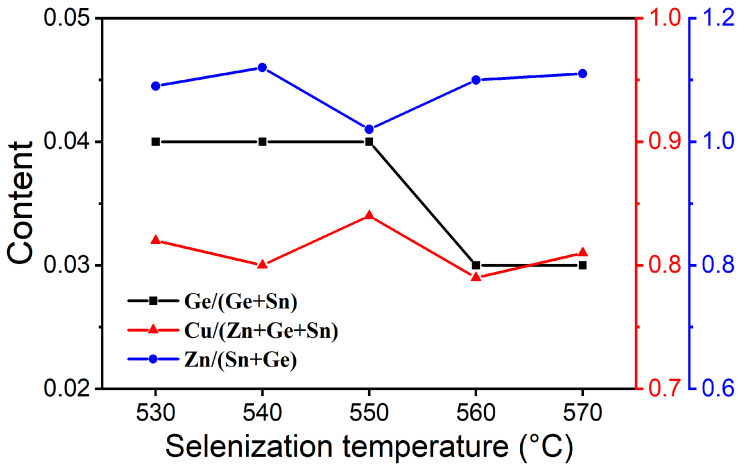
Cu/(Zn + Ge + Sn), Zn/(Sn + Ge) and Ge/(Ge + Sn) ratios of CZTGSSe films selenized at different temperatures.

Table 1 presents the elemental compositions of CZTGSSe films selenized at various temperatures, with all the values accompanied by the corresponding standard deviations derived from five EDS measurements. To better visualize the compositional trends, Figure 7 displays the variations in the Cu/(Zn + Ge + Sn), Zn/(Sn + Ge), and Ge/(Ge + Sn) ratios as a function of selenization temperature. All the samples exhibit a Cu-poor and Zn-rich stoichiometry, a composition known to suppress detrimental defects and thus enhance the crystalline quality and optoelectronic properties [26,27]. The Ge/(Ge + Sn) ratios of all the films are lower than those in the precursor solution and clearly depend on temperature: Ge volatilization is relatively weak at temperatures below 550 °C but becomes more pronounced above 550 °C. This Ge loss can be associated with the volatilization of Ge-containing species (including GeSe_2_) at relatively high temperatures [28].

**Figure 8:**
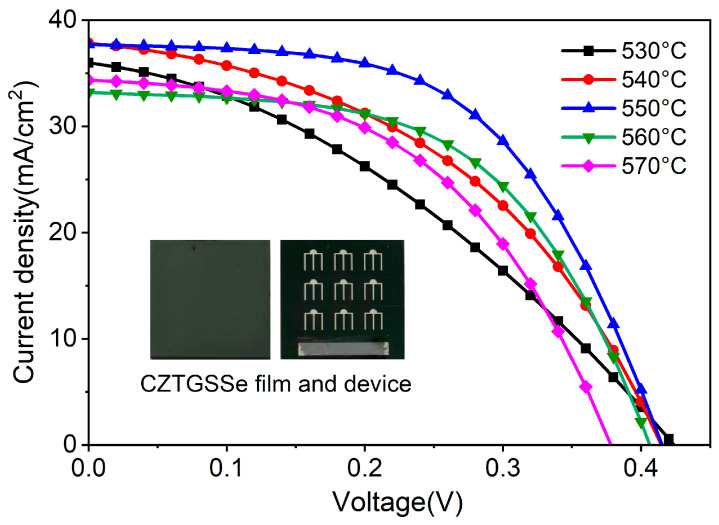
*J–V* curves showing CZTGSSe devices under different selenization temperatures.

The *J–V* characteristics of AM 1.5G-illuminated CZTGSSe solar cells are shown in Figure 8. Table 2 lists the photovoltaic parameters, and all the data are presented with the corresponding average values and standard deviations obtained from measurements on five independent cells. Typically, the peak PCE of the champion device synthesized at 550 °C is 8.69%, its *V*_OC_ is 0.42 V, the *J*_SC_ is 37.71 mA·cm^−2^, and the FF is 55.51%. This performance can be associated with the decreased series resistance (*R*_S_ = 15.33 Ω·cm^2^) and the increased shunt resistance (*R*_sh_ = 4935.61 Ω∙cm^2^), suggesting effective charge transport and the lowest parasitic loss. At temperatures of up to 550 °C, the device performance progressively increases, which is probably associated with the improved crystallinity, enhanced grain growth, and dense absorber layer generation, all of which promote carrier transport while suppressing bulk recombination [26]. In contrast, above 550 °C, the degradation of each critical photovoltaic parameter is related to increased surface roughness, decreased crystallinity, and Ge content loss, exacerbating interface and bulk recombination [17]. The above results revealed that 550 °C was the best selenization temperature for balancing optoelectronic performance and structural quality.

**Figure 9:**
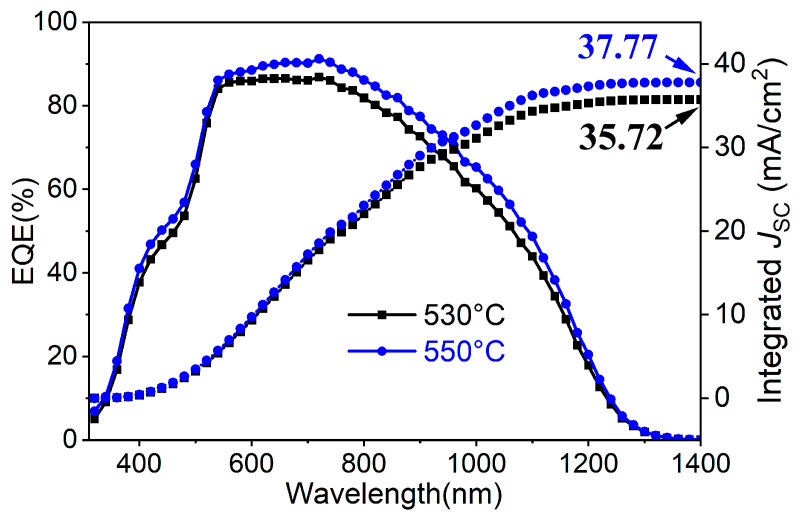
EQE spectra and integrated *J*_SC_ for CZTGSSe devices selenized at 530 °C and 550 °C.

The EQE spectra and integrated *J*_SC_ of CZTGSSe solar cells selenized at 530 °C and 550 °C are shown in Figure 9. Compared with the device selenized at 530 °C, the device selenized at 550 °C has a wider spectral response range and stronger photoresponse intensity. The integrated *J*_SC_ values of the CZTGSSe solar cells selenized at 530 °C and 550 °C from the EQE curves are 35.72 mA·cm^−2^ and 37.77 mA·cm^−2^, respectively, which are in good agreement with the *J*_SC_ values measured under standard AM1.5 illumination. Specifically, the enhanced performance of the 550 °C-selenized device in the short-wavelength region (<500 nm) is closely associated with the smoother surface of the absorber (RMS roughness = 52.3 nm). This low roughness can reduce surface defects, enabling conformal, uniform, and continuous CdS buffer layer coverage during chemical bath deposition—effectively passivating the CZTGSSe/CdS heterojunction and reducing interfacial recombination losses of near-surface short-wavelength-excited carriers [29]. The improved EQE within the mid-to-long wavelength region (500–1000 nm) is associated with a higher absorber quality at 550 °C, such as increased grain size, enhanced crystallinity, and adequate Ge addition, thereby suppressing bulk defects while improving carrier collection [30]. The steeper absorption profile within the long-wavelength region demonstrates a lower band-tail state density, indicating efficient Sn^4+^ substitution with Ge^4+^ and high lattice order, thus alleviating non-radiative recombination and band fluctuations [31]. Accordingly, the 550 °C-selenized device delivers the highest PCE of 8.69%. The high, broad EQE spectrum contributes directly to the large *J*_SC_ (37.71 mA·cm^−2^), whereas the low defect density and excellent interface quality yield a high FF (55.51%). These results establish a clear link between the EQE response and device performance, confirming that the device selenized at 550 °C achieves improved photon conversion efficiency and charge-transport efficiency.

**Figure 10:**
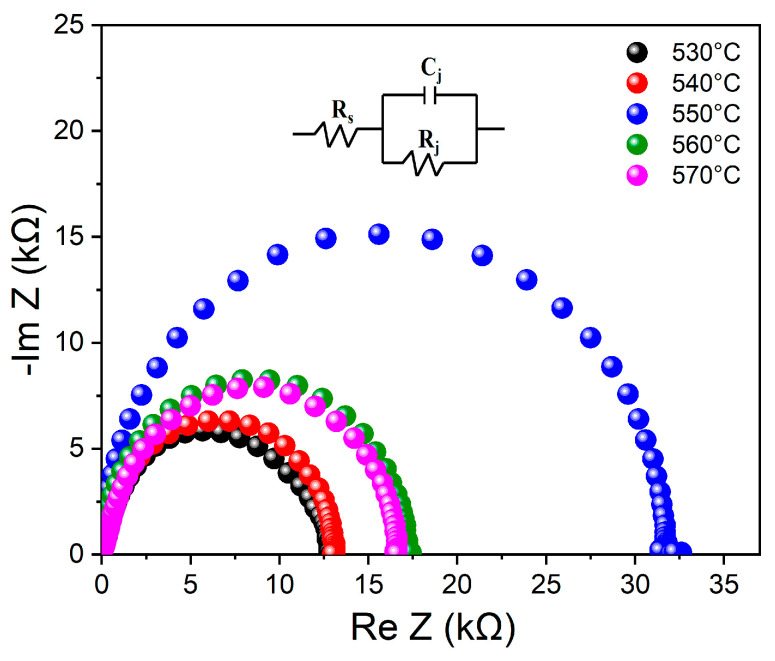
EIS results for the CZTGSSe devices at various selenization temperatures. Inset: Equivalent circuit schematic for the p-n junction within the device.

The Nyquist plots for the CZTGSSe devices detected away from light at zero bias at different selenization temperatures are shown in Figure 10. *R*_s_ corresponds to the real-axis intercept, which represents the total resistance encountered by charge carriers during transport (directly related to the impedance theory of polycrystalline materials), including the contact resistance, bulk resistance of the absorber layer, and grain boundary resistance. *R*_j_ is given by the diameter of the semicircular arc, reflecting the resistance against non-radiative recombination of electron–hole pairs at the heterojunction and in the bulk of the absorber layer. An equivalent circuit of the p-n junction is shown in the inset. The *R*_j_ and the corresponding junction capacitance (*C*_j_) values were obtained by fitting the data using Zview software (software version: 3.5c). *τ*_EIS_ was then calculated from the diffusion-recombination model according to Equation (2) [32,33].

*τ*_EIS_ = *R*_j_ × *C*_j_ (2)

Table 3 summarizes the EIS-derived parameters of CZTGSSe devices fabricated at various selenization temperatures. When the selenization temperature increases from 530 °C to 550 °C, *τ*_EIS_ increases significantly from 10.6 μs to 35.8 μs, *R*_j_ increases from 2.3 kΩ·cm^2^ to 5.9 kΩ·cm^2^, and *R*_s_ decreases markedly from 29.1 kΩ·cm^2^ to 8.6 kΩ·cm^2^. This trend fully aligns with the impedance theory of polycrystalline materials: the increased grain size (confirmed by SEM and AFM) reduces the grain boundary length, thereby decreasing the grain boundary contribution to *R*_s_. Moreover, the improved crystallinity and reduced defect density (from the XRD results) suppress non-radiative recombination, leading to an increase in *R*_j_. The concurrent increase in *τ*_EIS_ and *R*_j_, coupled with the decrease in *R*_s_, indicates suppressed interface recombination, decreased transport barriers, and improved carrier collection, which are consistent with the established interpretation of EIS data for kesterite solar cells [34]. This trend is consistent with the increased grain size, better crystalline quality, and smoother surface at 550 °C (according to XRD, SEM, and AFM characterizations), suggesting that selenization temperature-mediated structural optimization directly enhances the absorber’s electronic performance [35]. After the selenization temperature increases to above 550 °C, *τ*_EIS_ and *R*_j_ dramatically decrease, whereas *R*_s_ slightly increases. Such degradation is associated with Ge loss, film decomposition, increased surface roughness and decreased crystallinity at high temperatures, a phenomenon conforming to the temperature-mediated performance degradation documented for additional Ge-doped kesterite systems [36]. These factors jointly improve non-radiative recombination while suppressing charge transport. Overall, on the basis of the EIS results, 550 °C is the optimal selenization temperature for optimizing carrier dynamics and minimizing recombination losses in CZTGSSe solar cells.

**Figure 11:**
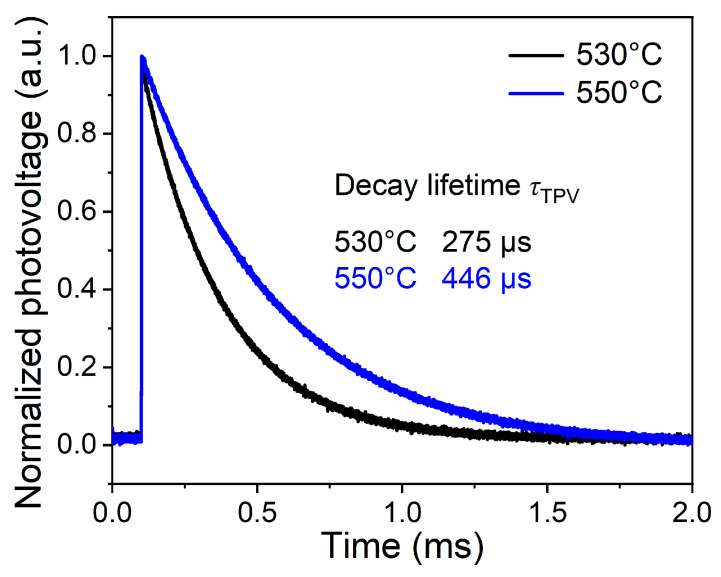
M-TPV decay spectra of CZTGSSe solar cells selenized at 530 °C and 550 °C.

To gain in-depth insights into the carrier transport mechanism and recombination dynamics of the devices, M-TPV measurements were performed to characterize the devices fabricated at selenization temperatures of 530 °C and 550 °C, as presented in Figure 11. The TPV decay lifetimes (*τ*_TPV_) of the devices at the two temperatures were determined by fitting the TPV decay curves with a single exponential model. The results show that the *τ*_TPV_ of the device selenized at 530 °C is 275 μs, whereas that of the device selenized at 550 °C is significantly extended to 446 μs, corresponding to an improvement of about 62% in the carrier recombination lifetime. This lifetime extension indicates that the optimal selenization temperature of 550 °C effectively reduces the number of harmful defects in the device and decreases the probability of nonradiative recombination events [37,38,39]. This is due to the combined effects of enhanced film crystallinity, an improved texture coefficient *T*_c_(112), the formation of large grains, a denser microstructure, and an optimized heterojunction interface quality at 550 °C.

Despite the optimal selenization temperature of 550 °C yielding a champion PCE of 8.69%, this value remains considerably lower than that of state-of-the-art kesterite devices (>15%). This efficiency gap stems from several interconnected constraints that synergistically limit device performance. First, the persistent fine-grained bottom layer induces severe back-contact recombination and introduces additional series resistance. This is directly reflected in the relatively high series resistance (*R*_S_ = 15.33 Ω·cm^2^) of our devices, which is substantially greater than that of record-efficiency kesterite systems (typically <5 Ω·cm^2^). This non-optimal carrier transport property not only restricts the fill factor (FF = 55.51%) but also impairs the bulk and interface carrier collection efficiency. Second, substantial Ge loss occurs during the selenization process. This not only compromises the intended defect suppression effect but also leads to incomplete bandgap engineering. Third, residual cation disorder in the kesterite lattice and non-ideal CZTGSSe/CdS heterojunction quality jointly contribute to a relatively high *V*_OC,def_. This is reflected in the moderate *V*_OC_ of 0.42 V, which is lower than the *V*_OC_ values of high-efficiency kesterite devices (>0.5 V) and indicates unaddressed non-radiative recombination losses in both the bulk and interfacial regions. Collectively, these factors synergistically limit the device performance, underscoring that while selenization temperature optimization is critical, further breakthroughs require synergistic strategies—including interface engineering, alkali metal doping, and refined selenization control—to address the inherent limitations of the kesterite system.

## 4. Conclusions

The present work utilized a green n-butylammonium butyrate-based solution approach to synthesize low-concentration Ge-doped (Ge/(Ge + Sn) = 0.05) CZTGSSe absorber layers alongside solar cells. According to our findings, the selenization temperature (530–570 °C) significantly regulated the microstructure and photovoltaic performance. Typically, 550 °C is the best selenization temperature, yielding the best CZTGSSe absorber with larger uniform grains, better crystalline quality, efficient Ge^4+^ retention and decreased surface roughness. These structural properties inhibit non-radiative recombination and carrier transport resistance, resulting in a device PCE of 8.69%. Our results indicate that the selenization temperature is an essential process parameter for optimizing the performance of low-concentration Ge-doped kesterite solar cells, which provides an important complement to the current emphasis on compositional doping ratios.

Despite these advances, the system still possesses inherent limitations: the 8.69% power conversion efficiency remains considerably lower than that of state-of-the-art kesterite (>15%) and CIGS/CdTe devices; the persistent fine-grained layer induces severe back-contact recombination; and residual cation disorder, Ge volatilization, and non-ideal CZTGSSe/CdS heterojunction quality continue to constrain device performance. Future research should address these challenges through synergistic optimization of the following strategies: (i) interface passivation engineering (e.g., hydrogen-treated CdS or (Zn,Sn)O buffer layers); (ii) synergistic alkali metal doping (e.g., Na/Li) to promote grain growth and inhibit Ge volatilization; and (iii) refined selenization control (precise Se partial pressure and ramping profiles) to eliminate the fine-grained layer. The above strategies provide a systematic pathway for overcoming the performance bottlenecks of Ge-doped kesterite solar cells.

## Figures and Tables

**Table 1:** EDS data for CZTGSSe films selenized at different selenization temperatures.

Selenization Temperatures	Composition (at.%) in Films						Compositional Ratio in Films		
	Cu	Zn	Sn	Ge	S	Se	Cu/(Zn + Ge + Sn)	Zn/(Sn + Ge)	Ge/(Ge + Sn)
530 °C	21.5 ± 1.21	13.7 ± 0.86	12.1 ± 0.37	0.5 ± 0.07	4.7 ± 0.48	45.0 ± 2.26	0.82	1.09	0.04
540 °C	20.5 ± 0.61	13.5 ± 0.44	11.6 ± 0.23	0.5 ± 0.04	4.3 ± 0.35	46.8 ± 0.85	0.80	1.12	0.04
550 °C	20.5 ± 1.16	12.3 ± 0.83	11.6 ± 0.30	0.5 ± 0.05	4.1 ± 0.43	48.4 ± 2.06	0.84	1.02	0.04
560 °C	19.0 ± 1.12	12.7 ± 0.53	11.2 ± 0.26	0.3 ± 0.04	5.6 ± 0.37	48.0 ± 2.15	0.79	1.10	0.03
570 °C	21.0 ± 0.98	13.6 ± 0.43	11.9 ± 0.36	0.4 ± 0.04	3.3 ± 0.30	47.0 ± 1.12	0.81	1.11	0.03

**Table 2:** Photovoltaic parameters for CZTGSSe devices at different selenization temperatures.

Selenization Temperatures	*V*_OC_ [V]	*J*_SC_ [mA/cm^2^]	*R*_s_ [Ω∙cm^2^]	*R*_sh_ [Ω∙cm^2^]	FF [%]	PCE [%]
530 °C	0.42 ± 0.02	35.99 ± 2.88	35.06 ± 2.03	350.84 ± 34.32	35.65 ± 3.35	5.44 ± 0.32
540 °C	0.41 ± 0.01	37.85 ± 2.50	18.92 ± 1.80	422.10 ± 41.00	44.34 ± 3.00	6.96 ± 0.86
550 °C	0.42 ± 0.02	37.71 ± 1.95	15.33 ± 2.54	4935.61 ± 303.26	55.51 ± 2.29	8.69 ± 0.80
560 °C	0.41 ± 0.02	33.19 ± 1.62	17.19 ± 2.61	1331.28 ± 152.07	55.19 ± 2.82	7.46 ± 0.97
570 °C	0.38 ± 0.01	34.35 ± 2.30	17.49 ± 1.70	962.33 ± 103.14	49.51 ± 3.20	6.43 ± 0.51

**Table 3:** EIS-derived parameters of CZTGSSe devices with various selenization temperatures.

Selenization Temperatures	*R*_s_ (Ω∙cm^2^)	*R*_j_ (kΩ∙cm^2^)	*C*_j_ (nF)	*τ*_EIS_ (µs)
530 °C	29.1	2.3	4.6	10.6
540 °C	27.5	2.4	6.0	14.6
550 °C	8.6	5.9	6.0	35.8
560 °C	9.1	3.2	5.4	17.3
570 °C	9.3	3.0	6.2	18.3

## Data Availability

The original contributions presented in this study are included in the article. Further inquiries can be directed to the corresponding authors.
